# Current Evidence Supporting the Role of miRNA as a Biomarker for Lung Cancer Diagnosis Through Exhaled Breath Condensate Collection: A Narrative Review

**DOI:** 10.3390/life15050683

**Published:** 2025-04-22

**Authors:** Paolo Albino Ferrari, Cosimo Bruno Salis, Antonio Macciò

**Affiliations:** 1 Division of Thoracic Surgery, Oncology Hospital “A. Businco”, Azienda di Rilievo Nazionale ed Alta Specializzazione “G. Brotzu”, Via Jenner Snc, 09121 Cagliari, Italy; 2 Department of Medicine, Surgery and Pharmacology, University of Sassari, Viale San Pietro 43a, 07100 Sassari, Italy; cosimobrunosalis@gmail.com; 3 Department of Surgical Sciences, University of Cagliari, SS. 554, km 4500, 09042 Monserrato, Italy; antoniomaccio56@gmail.com

**Keywords:** lung cancer, miRNA, exhaled breath, next generation sequencing, biomarker

## Abstract

Lung cancer, the leading cause of cancer-related mortality, has brought exhaled breath condensate (EBC) into focus as a promising non-invasive sample for detecting molecular biomarkers, particularly microRNAs, which regulate gene expression and contribute to tumorigenesis. Ten key studies encompassing approximately 866 subjects consistently demonstrated distinct patterns of miRNA dysregulation in lung cancer. Notably, several reported panels achieved diagnostic sensitivity and specificity exceeding 75% through the identification of distinct miRNA signatures in EBC, with oncogenic miRNAs (e.g., miR-21) upregulated and tumor-suppressor miRNAs (e.g., miR-486) downregulated in lung cancer patients. Analytical advancements, including next-generation sequencing (NGS), have improved miRNA detection sensitivity and specificity, addressing prior limitations of low yield and variability. NGS enabled the identification of novel miRNAs and proved especially effective in overcoming the low RNA yield associated with EBC samples. However, challenges persist regarding standardization of collection, sample dilution, and potential contamination. Moreover, the reproducibility of miRNA signatures across diverse patient populations remains a critical issue. Large-scale, multicenter validation studies are needed to establish robust diagnostic algorithms integrating EBC-derived miRNAs with existing clinical tools. The potential of EBC miRNA profiling to support current screening strategies could significantly improve early lung cancer detection and patient outcomes. Nevertheless, its clinical transition requires further methodological optimization and biomarker validation. This review critically evaluates current evidence on miRNA detection in EBC for lung cancer diagnosis.

## 1. Introduction

The National Institute of Health defines biomarkers as “characteristics that are objectively measured and evaluated as indicators of normal biological processes, pathogenic processes, or pharmacologic responses to a therapeutic intervention” [1]. To be used in clinical settings, a biomarker should improve patient outcomes, address an unmet clinical need, and provide advantages over standard practice [2].

Lung cancer is the leading cause of cancer-related mortality worldwide, with a 5-year survival rate of less than 15% [3,4,5]. This poor prognosis is primarily because most cases are diagnosed at an advanced stage when treatment options are limited. Early detection is further complicated by the nonspecific nature of initial symptoms, which often overlap with those of other chronic respiratory diseases, such as COPD [6].

The identification of clinically useful biomarkers for lung cancer detection—both at early and metastatic stages—is an urgent medical need. In 2022 alone, lung cancer accounted for approximately 2.4 million new cases and 1.8 million deaths globally [7]. Although tissue biopsy is the current gold standard for lung cancer diagnosis and genetic profiling, it presents limitations such as invasiveness, low patient compliance, and challenges in accessing certain tumor sites [8]. Liquid biopsy (LB) has emerged as a non-invasive alternative, enabling the detection of tumor-derived components, such as circulating tumor cells and cell-free nucleic acids, in biofluids like blood, saliva, and urine [9,10]. Blood remains the most common source for LB. However, fluids anatomically closer to the lungs, such as sputum and exhaled breath condensate (EBC), may offer more direct insights into tumor-related molecular alterations [11].

Among these alternative sources, EBC has gained attention as a promising non-invasive sample for biomarker discovery. EBC, derived from airway lining fluid, contains both volatile and non-volatile molecules such as nitric oxide, organic acids, proteins, and surfactants [12,13,14]. Its simplicity and non-invasiveness make EBC a valuable tool for investigating respiratory diseases, including lung cancer [15,16].

In recent years, research has increasingly focused on micro ribonucleic acids (miRNAs) in EBC due to their crucial role in tumorigenesis through regulating cell cycle progression, metastasis, and angiogenesis [17]. Despite the growing interest in circulating miRNAs in plasma and sputum, relatively few studies have explored EBC as a source of miRNA biomarkers. Due to its non-invasive collection method and anatomical proximity to the tumor site, EBC offers a unique and underutilized opportunity for direct lung-specific molecular profiling.

This review aims to consolidate the current evidence supporting the utility of miRNAs in EBC for lung cancer detection, diagnosis, and prognosis value.

## 2. MicroRNAs as a Diagnostic Biomarker of Lung Cancer

MiRNAs are small, non-coding RNA molecules with a length of around 21 nucleotides and play an important role in post-transcriptional gene regulation [1]. They are involved in several biological processes, including cell differentiation, proliferation, and apoptosis, so they have been identified as possible biomarkers for early diagnosis and prognosis in different diseases [18,19]. Compared to healthy individuals, miRNAs are frequently either upregulated or downregulated in lung cancer [2,20,21], as well as in various other lung diseases such as asbestosis [3], cystic fibrosis [4], or chronic lung infections due to Pseudomonas aeruginosa colonization [5].

MiRNAs can be collected from various biological sources, including tissues or single-cell samples, which are used to isolate these molecules from specific cell types and biofluids (e.g., plasma, serum, and saliva) [6,7,8,22]. In recent years, the extraction of miRNAs from extracellular vesicles derived from EBC has received substantial scientific interest and relevance [23].

There are several miRNA profiling approaches, each with its own set of advantages and disadvantages. Quantitative real-time protein chain reaction (qRT-PCR) is noted for its excellent sensitivity and specificity in measuring known miRNAs, although its use is limited to specialized investigations [9]. Nonetheless, this approach has played a critical role in scientific research comparing miRNA expression levels in persons with lung disease and healthy control groups, offering vital insights into illness-related miRNA dysregulation [10]. Microarrays offer cost-effective, high-throughput profiling but lack sensitivity and specificity [24,25]. In recent years, Next-Generation Sequencing (NGS) has transformed miRNA profiling by providing unparalleled sensitivity and specificity, allowing for exhaustive investigation of known and new miRNAs. Despite its expensive cost and complexity, NGS remains the gold standard for advanced research [11].

### 2.1. miRNA’s Role in Lung Cancer Detection

The diagnosis and prognosis of lung cancer can be accurately determined by analyzing specific miRNA expression patterns, known as miRNA signatures [26,27]. Numerous studies have identified unique miRNA signatures in lung cancer patients, demonstrating their high sensitivity and specificity for diagnostic purposes [28,29,30,31,32].

The analysis of miRNA expression has emerged as a powerful tool for identifying the unknown origin of metastatic tissues. For instance, Rosenfeld and colleagues conducted a comprehensive miRNA study on 22 of the most common solid tumours, leading to the development of a 48-miRNA classifier capable of pinpointing the origin of cancers of unknown primary with 81% sensitivity [33].

The dysregulation of miRNAs can be observed at various stages of tumorigenesis, from initiation to progression, providing real-time insights into cancer dynamics [34]. These findings fuel optimism for developing minimally invasive early lung cancer detection strategies by evaluating circulating cell-free miRNAs.

The overexpression of miRNA-21 in sputum, a well-recognized anti-apoptotic factor regulated by epidermal growth factor (EGF), has been shown to effectively distinguish non-small cell lung cancer (NSCLC) patients from cancer-free individuals [35]. Additionally, distinct miRNA signatures with diagnostic and prognostic values have been identified in sputum samples [36]. A meta-analysis by Zheng et al. revealed that an integrated panel combining plasma and sputum-derived miRNAs enhances the sensitivity and specificity of lung cancer diagnosis [37]. Notably, this panel’s diagnostic performance was independent of histological subtype, cancer stage, patient age, sex, and ethnicity [38].

Several miRNAs have been proposed as potential biomarkers for NSCLC diagnosis and prognosis. MiR-23a and miR-let7i, for example, may hold clinical utility in diagnosing NSCLC [39], while miR-21-5p, miR-150, miR-210, and miR-1290 have been identified as promising early diagnostic and prognostic markers [40]. Similarly, a panel of miRNAs, including miR-30a-3p, miR-30b-5p, miR-30c-5p, miR-34a-5p, and miR-4286, has shown potential as a novel biomarker set for lung cancer detection and prognosis [41]. Another study identified a five-miRNA panel (hsa-miR-31, hsa-miR-34c, hsa-miR-196b, hsa-miR-653, and hsa-miR-891a) as a promising biomarker set for lung cancer diagnosis and prognosis [42]. Moreover, Yu-Long Zhao et al. reported serum miR-205-5p as a novel diagnostic biomarker for lung cancer [43], and miR-3182 has also been proposed as a potential diagnostic marker [44].

The application of miRNA signatures in cancer screening has progressed to preclinical and clinical testing. One notable effort, the mir-Test, aims to establish a sensitive, non-invasive method for early lung cancer detection, particularly in high-risk populations such as heavy smokers over 50 [45]. However, for miRNA biomarkers to be effectively implemented in clinical practice, large-scale studies with standardized protocols, platforms, and data-analysis methodologies are essential to ensure reproducibility and diagnostic accuracy [46].

Despite these promising findings, there remains a significant gap in the literature regarding the validation of EBC-derived miRNA biomarkers across diverse populations. Most existing studies are limited in sample size and geographic scope, and lack multi-center validation, which hinders the generalizability of their diagnostic value [17].

### 2.2. miRNA Profiling for Lung Cancer Subtype Classification

Considering the heterogeneity of lung cancer, miRNA expression profiles can also aid in the classification of its subtypes. Researchers have demonstrated that miRNA signatures are highly specific to histological subtypes, allowing for precise discrimination among different forms of lung cancer. Several studies have validated the robustness of miRNA profiling in distinguishing between subtypes, a crucial factor in guiding treatment decisions, particularly between squamous cell carcinoma and adenocarcinoma, which originate from distinct lung cell types and require different therapeutic approaches [47,48,49,50]. Furthermore, miRNA expression analysis can differentiate primary lung tumours from metastatic lesions originating from other organs. Notably, miR-182 is significantly overexpressed in primary lung tumours, whereas miR-126 is more prevalent in lung metastases derived from other tissues [51].

### 2.3. miRNA Oncogenic Family

The miR-17-92 cluster, located at 13q31.3 within the C13orf25 gene, consists of seven different miRNAs: miR-17-3p, miR-17-5p, miR-18a, miR-19a, miR-20a, miR-19b-1, and miR-92a [52]. This cluster is frequently overexpressed in lung cancer, particularly in small-cell lung cancer [53]. Overexpression of the miR-17-92 cluster inhibits the expression of E2 promoter-binding factor 1, hypoxia-inducible factor 1α, and phosphatase and tensin homolog (PTEN), thereby promoting cell proliferation and tumour progression [54,55].

MiR-21 is a widely recognized oncogenic miRNA upregulated in various cancers. It promotes tumorigenesis and inhibits apoptosis by indirectly activating the rat sarcoma virus (RAS)/mitogen-activated protein kinase kinase (MEK)/extracellular signal-regulated kinase-signaling pathway. The increased expression of miR-21 results in the downregulation of key tumour suppressor genes, including PTEN, programmed cell death protein 4, and tropomyosin alpha-1, thus fostering cell proliferation and migration while preventing apoptosis [56,57]. Recent studies have also shown that miR-21-5p can suppress mothers against decapentaplegic homolog 7 expression in lung cancer cells, promoting cell proliferation, migration, and invasion [57]. Additionally, in vitro research indicates that miR-21 upregulation in lung fibroblasts leads to their transdifferentiation into cancer-associated fibroblasts, contributing to enhanced cancer progression [58].

MiR-31 is another oncogenic miRNA involved in lung cancer. It inhibits cell growth and tumorigenicity by directly repressing the tumour suppressor large tumour suppressor kinase 2 (LATS2) and protein phosphatase 2 regulatory subunit B α (PPP2R2A), establishing a novel regulatory network in lung cancer via the miR-31-LATS2-PPP2R2A pathway [59]. Overexpression of miR-31 is frequently observed in human lung adenocarcinoma and correlates with decreased survival rates. Inducing miR-31 has been shown to promote lung hyperplasia, adenoma formation, and lung adenocarcinoma progression. Moreover, miR-31 suppresses the expression of six key RAS/MEK pathway regulators, thereby facilitating tumorigenesis [60].

MiR-221 and miR-222 are critical regulators in lung cancer development and progression. These miRNAs promote tumorigenesis by repressing PTEN and the tissue inhibitor metalloproteinase 3, enhancing cell proliferation and migration [61]. Additionally, miR-221 and miR-222 are modulated by EGF and mesenchymal-epithelial transition (MET) proto-oncogene receptors in tyrosine kinase inhibitor-resistant NSCLC, further contributing to cancer progression.

### 2.4. Tumour-Suppressing miRNAs

The miR-200 family is well-established as a key regulator of epithelial-to-mesenchymal transition, characterized by the loss of E-cadherin-mediated cell adhesion and increased cellular motility, which facilitates tumour invasion and metastasis [62]. miR-200 targets the transcriptional repressors of E-cadherin, specifically the zinc finger E-box-binding homeobox (ZEB1) and ZEB2 proteins. Thus, the upregulation of miR-200 results in E-cadherin overexpression and the reduction of lung cancer cell motility [63].

MiR-206 is markedly downregulated in clinical specimens of squamous cell lung carcinoma. It is thought to act as a tumour suppressor by modulating oncogenic pathways, such as MET and EGFR, by downregulating their signaling. Similarly, the expression of miR-133b is reduced in cancer cells, which is considered a tumour suppressor [64,65].

The Let-7 miRNA family, initially discovered in Caenorhabditis elegans, is critical in regulating cell fate and was the first miRNA identified in humans. It has been shown that an overexpression of Let-7 in the A549 lung cancer cell line induces cell cycle arrest and inhibits cell growth [66,67]. Let-7 represses the expression of several oncogenes, including RAS, myelocytomatosis oncogene (a master cell cycle regulator), and high mobility group AT-hook 2, all contributing to lung cancer cell proliferation. Additionally, Let-7 suppresses cyclin-dependent kinase 6 (CDK6), and a reduced expression of CDK6 promotes cell cycle progression [68]. Furthermore, Let-7 directly inhibits endoribonuclease, suggesting a role in regulating the broader production of miRNAs [69]. The Let-7 family is frequently deleted from lung cancer and is pivotal in controlling cancer progression [70].

In response to DNA damage, the tumour protein P53 gene is activated, leading to the induction of the miR-34 family, which regulates cell cycle arrest and apoptosis in cancer cells [71]. In lung cancer, the downregulation of miR-34 leads to the upregulation of target genes such as MET, B-cell lymphoma (Bcl2), and platelet-derived growth factor receptor (PDGFR) [72,73,74]. Reduced expression of miR-34 promotes cell proliferation by upregulating MET and Bcl2. Furthermore, miR-34-mediated downregulation of PDGFR inhibits tumorigenesis while enhancing the tumour necrosis factor-related apoptosis-inducing ligand in lung cancer [74].

## 3. Exhaled Breath Condensate

EBC analysis represents an emerging, non-invasive approach for detecting biomarkers primarily derived from the lower respiratory tract. EBC is obtained during tidal breathing through the cooling and condensation of exhaled aerosol [75]. This technique is unique among pulmonary function assessments as it enables the identification of molecular pathways that reflect airway epithelial physiology [76]. A key advantage of EBC analysis is its non-invasive, patient-friendly collection method, which requires only spontaneous respiration [77], in contrast to more invasive and technically demanding procedures such as bronchoalveolar lavage (BAL) [78], as summarized in Table 1.

Extensive literature has focused on detecting inflammatory mediators in EBC, offering insights into chronic airway diseases, including COPD, asthma, and cystic fibrosis [75,79]. However, recent methodological advancements have expanded the scope of EBC analysis to include metabolomic [80], proteomic [81,82], and genomic [83] profiling of exhaled breath to facilitate early diagnosis not only of respiratory pathologies [81,83] but also of systemic diseases [84,85,86].

There is a clear and growing trend in biomedical research toward developing non-invasive, patient-centric respiratory diagnostic techniques. Additionally, the volume of original research exploring the potential clinical applications of EBC analysis has increased substantially, employing diverse methodological approaches [87].

### 3.1. EBC Collection Systems

Compared to blood sampling, nasal, and BAL in the context of respiratory diseases, EBC collection enables the assessment of a broader surface area of the respiratory tract lining fluid. This approach preserves the physiological integrity of the airway epithelium, minimizing potential perturbations associated with invasive procedures [88,89].

EBC collection requires only tidal breathing by the subject, facilitated through a controlled cooling system for exhaled air. The procedure usually involves applying a nose clip to prevent nasal airflow interference. The subject then breathes naturally for approximately 10 min through a specialized mouthpiece equipped with a saliva trap and a one-way valve. This configuration ensures that the exhaled air is directed through a Teflon or polypropylene tube housed within a cooling system, where aerosolized respiratory droplets undergo condensation to form EBC [75,90].

Various custom-built EBC collection devices have been described in the literature, generally adhering to two fundamental designs: a Teflon tube submerged in an ice-filled container to achieve air condensation and a double-layered glass chamber where exhaled air undergoes condensation between the two thermally regulated layers [75]. In addition to these laboratory-assembled systems, several commercially available devices, including ECoScreen, TurboDeccs, RTube, and the Anacon glass condenser, have been developed based on these principles, offering standardized and reproducible sample collection methodologies with relative pro and contra (Table 2). Some EBC collection systems offer optional modules that allow for real-time measurement of exhaled volume. These features can support procedural standardization by enabling normalization of biomarker concentrations to the total exhaled volume, thereby improving cross-study comparability.

Among these, the RTube is FDA-cleared for clinical use and remains one of the most widely used systems in research and pilot diagnostic settings. Its FDA clearance underscores its potential for clinical translation, although its application currently remains mostly limited to research environments [61].

### 3.2. EBC Dilution and Sample Contamination

EBC is a matrix predominantly composed of water vapour [94], leading to significant biomarker dilution, often approaching the lower sensitivity thresholds of analytical assays. Since EBC exhibits more significant dilution than ALF [95], it is imperative to establish a reliable dilution indicator that accurately reflects biomarker concentrations within the airways. According to the literature [96], an ideal dilution marker should possess a well-characterized and stable plasma concentration, exhibit high membrane diffusivity, and be exogenous to the respiratory tract.

No universally accepted gold standard for EBC dilution correction has been established. However, several surrogate indicators have been proposed, including urea [97,98,99,100], total protein content [100], total cation concentration (Na^+^, K^+^, Ca^2+^, Mg^2+^), and the conductivity of lyophilized and vacuum-evaporated samples [97,98].

A major limitation to the clinical adoption of EBC-based biomarkers lies in the significant inter-patient and intra-patient variability of EBC content, which may result from differences in breathing patterns, collection time, device types, and environmental conditions. Furthermore, the lack of standardized protocols for sample collection, RNA extraction, and downstream analysis hampers reproducibility and cross-study comparability. International efforts to establish consensus guidelines for EBC handling and analysis are urgently needed to move this field forward [75,97].

Since the primary objective is to analyze compounds originating from the lower respiratory tract, it is essential to minimize sample contamination from mediators and proteins derived from saliva, the oral cavity, and the upper airways [101]. Various methodologies have been implemented to prevent contamination, including using saliva traps, swan neck-shaped collection tubes, pre-collection mouth rinsing with 4.5% sodium bicarbonate solution, and encouraging subjects to perform periodic swallowing during sampling [75,102].

The quantification of salivary amylase in EBC has been proposed to verify the absence of salivary contamination post-collection. When appropriate precautions are implemented to minimize salivary contamination, such as using saliva traps and instructing subjects to swallow periodically during collection, amylase concentrations in EBC are significantly reduced, typically found at levels approximately 10,000 times lower than those in saliva [97,103].

### 3.3. EBC Sample Preservation and Analysis Methods

Collected EBC samples should be analyzed immediately or stored at −70 °C to maintain molecular integrity. The targeted chemical compounds’ stability profile dictates the maximum storage duration. Repeated freeze–thaw cycles must be strictly avoided, as they contribute to thermolabile analyte degradation and potential loss [104].

Although enzyme-linked immunosorbent assay (ELISA) kits are widely used, they often lack the sensitivity and specificity needed for the extremely low analyte concentrations found in EBC [105,106,107]. Thus, while ELISA and other immunoassay-based techniques, such as radioimmunoassay, immune sensors, and multiplex immunoassay, are frequently employed in EBC studies [107], their findings typically require confirmation through high-sensitivity platforms like mass spectrometry (MS) or high-performance liquid chromatography (HPLC) [108].

Proteomic and metabolomic analysis advancements have introduced high-resolution techniques such as two-dimensional polyacrylamide gel electrophoresis [108], followed by chromatographic proteomic microanalysis, providing both qualitative and quantitative insights into the EBC profile. Contemporary chromatographic methodologies include gas chromatography (GC), HPLC, and MS, along with hybrid analytical techniques such as liquid chromatography-MS, GC-MS [109], HPLC-MS [110], chromatography-differential mobility spectrometry [111], and electrospray ionization-differential mobility spectrometry [112].

## 4. miRNA Identification and Its Clinical Value in EBC Sample

Three principal methodologies are employed for quantifying miRNA expression levels: qRT-PCR, microarray hybridization, and, more recently, NGS [113,114,115]. High-throughput NGS technology has demonstrated superior sensitivity compared to qRT-PCR and microarray techniques. NGS offers enhanced cost efficiency, significantly broader sequence coverage and depth, and the capacity to identify novel, previously uncharacterized miRNAs [116], surpassing conventional methodologies’ capabilities and driving significant advancements in the EBC field due to specific workflow methodologies (Table 3).

Due to the well-known issues of repeatability, specificity, and sensitivity, finding the most suitable specimen for analyzing miRNA biomarkers is challenging. The EBC sampling would be less invasive, rapid, cost-effective, and easily repeatable [117].

EBC sampling has also provided recent progress in identifying other biological macromolecules, such as lipids, proteins, DNA, and oxidative stress markers [118], extending its potential applications in clinics, prevention, and epidemiology. The low yield of miRNA from the EBC is a significant obstacle, which was successfully overcome in two studies using the NGS technology [117,118,119]. Also, its potential for discovering previously unidentified miRNAs [116] would make it more suitable for future projects to detect new molecular signatures as early biomarkers of profession-related health effects such as asbestos exposure.

Based on these assumptions, Cherchi et al. conducted a preliminary test on male volunteers to compare the EBC miRNA profile to that in the plasma using two sequencing platforms that were different in cost and performance [93].

**Table 3:** MiRNA Detection from EBC via NGS—Methodological Overview.

Study	EBC Collection Method	EV Isolation (If Any)	RNA Extraction Method	Library Preparation	Sequencing Platform	Bioinformatic Workflow
Cherchi et al. (2023) [93]	TurboDeccs (Medivac Srl, Parma, Italy), −5 °C, 15–20 min, 100 L target	Not applied	miRNeasy Serum/Plasma Advanced Kit (Qiagen, Venlo, Netherlands), 200 µL input	QIAseq miRNA Library Kit with UMIs	Illumina MiSeq & HiSeq3000	Cutadapt trimming, Bowtie mapping, miRBase v21, UMI deduplication
Mithcell et al. (2024) [120]	RTube (Respiratory Research Inc., Austin, TX, U.S.), 10 min tidal breathing, 1.5–2 mL	Differential ultracentrifugation + EV-CATCHER	miRNeasy Serum/Plasma Kit (Qiagen), 100 µL EVs	Custom PAGE-purified small RNA library	Illumina HiSeq 2500	RNAworld trimming, DESeq2/edgeR via Bioconductor
Stachowiak et al. (2020) [119]	TurboDeccs (Medivac Srl, Parma, Italy), −5 °C, 3 mL collected	Exosomes from EBC using miRCURY kit (Qiagen)	RNeasy mini kit + MinElute columns (Qiagen)	TruSeq Small RNA Library Kit (Qiagen)	Illumina MiniSeq	BaseSpace for NGS data; qPCR validation (miR-486-5p, miR-223, etc.)

miRNA: micro ribonucleic acid; EBC: Exhaled Breath Condensate; NGS: Next-Generation Sequencing; UMI: Unique Molecular Indexes; EV: Extracellular Vesicles; qPCR: quantitative protein chain reaction.

### 4.1. miRNAs as Potential EBC Biomarkers for Lung Cancer Diagnosis

Growing evidence supports the role of miRNAs in EBC as promising diagnostic biomarkers for lung cancer. One of the pioneering studies, conducted by Mozzoni et al. [121], analyzed EBC samples from 54 patients with NSCLC and 46 healthy controls. The cancer group included 37 adenocarcinoma and 17 squamous cell carcinoma cases, primarily diagnosed at early stages. The study focused on two miRNAs with opposing functions: miR-21, an oncogenic miRNA, and miR-486, a tumor-suppressing miRNA. The findings revealed a marked upregulation of miR-21 and a concurrent downregulation of miR-486 in NSCLC patients, which were further validated in plasma and tissue samples.

It would be pertinent to indicate whether any of the reported miRNA signatures—particularly those involving miR-21 and Let-7 family members—have advanced to clinical validation stages. For example, the miR-Test developed by Marzi et al. has undergone preliminary clinical evaluation for early lung cancer detection in high-risk populations, representing a step toward translational application of circulating miRNA-based diagnostics [45].

### 4.2. Validation of miRNA Biomarkers in EBC

Further research has reinforced these findings. A case-control study analyzed EBC samples from 15 lung cancer patients (pathologically confirmed and untreated) and 15 high-risk individuals using the EcoScreen condenser (Jaeger, Hochberg, Germany). Quantitative real-time PCR detected a significant upregulation of miRNA-155 in lung cancer patients, aligning with previous studies identifying miRNA-155 as a critical biomarker with diagnostic and prognostic potential [122].

It may be appropriate to emphasize that miR-21 and miR-155 have demonstrated consistent upregulation across multiple independent studies, suggesting their robustness as candidate biomarkers for lung cancer diagnosis.

A similar investigation through qRT-PCR described the upregulation of miR-21 in the NSCLC patient group compared to the healthy control group, based on miRNA extraction from EBC collection [123].

Another study by Chen et al. [124] examined the association between Let-7 miRNA expression and NSCLC, using reverse transcription-quantitative PCR on 180 samples from 30 NSCLC patients and 30 healthy controls. The results demonstrated significantly lower Let-7 levels in NSCLC patients, which further decreased with disease progression and lymph node metastasis. Similarly, a study conducted in a Chinese cohort assessed the clinical relevance of miR-186 and interleukin-1β in NSCLC [125], revealing a significant reduction in miR-186 levels in both serum and EBC samples, particularly in early-stage cases.

### 4.3. Genome-Wide Profiling of miRNAs in Lung Cancer

Genome-wide profiling has expanded the understanding of miRNA expression in lung cancer. Pérez–Sánchez et al. [126] employed GeneChipR miRNA 4.0 arrays to analyze genome-wide miRNA expression in 42 subjects (21 lung cancer patients and 21 healthy controls). The study identified nine upregulated and three downregulated miRNAs in lung cancer EBC samples. Among these, miR-4507 exhibited the highest diagnostic accuracy, followed by miR-6777-5p and miR-451a. Additionally, three miRNAs (miR-4529-3p, miR-8075, and miR-7704) effectively differentiated adenocarcinoma from squamous cell carcinoma, with 100% specificity and 88% sensitivity. Furthermore, a distinct miRNA signature distinguished stage IV NSCLC from earlier stages.

These results should be interpreted cautiously, as the identified miRNAs are derived from limited sample sizes and currently lack external validation in independent cohorts. Their diagnostic utility remains preliminary.

Another study profiling 754 unique miRNAs in EBC from 14 early-stage lung adenocarcinoma patients and nine healthy individuals identified 11 miRNAs associated with adenocarcinoma. Among these, miR-597-5p and miR-1260a showed significant elevation in adenocarcinoma patients compared to controls and were also detected in pleural mesothelioma, albeit predominantly expressed in lung adenocarcinoma [127].

A comprehensive summary of the studies investigating miRNA-based lung cancer discrimination in EBC is presented in Table 4.

### 4.4. Next-Generation Sequencing and Emerging Biomarkers

Recent studies employing NGS have further validated miRNA deregulation in lung cancer [29]. Rai et al. analyzed miRNA expression in EBC from 30 lung cancer patients, identifying 78 differentially expressed miRNAs [128]. Six miRNAs were selected for further validation, with Let-7i, miR-449c, and miR-31-3p demonstrating significant upregulation in lung cancer patients. Let-7i exhibited 75% sensitivity and 95% specificity, while miR-449c and miR-31-3p displayed similarly high diagnostic performance.

A recent clinic-based case-control study by Shi et al. (166 NSCLC cases, 185 controls) confirmed that individual exhaled miRNAs (miR-21, miR-33b, miR-212) provided case-control discrimination through logistic regression models. More advanced random forest models suggested modest improvements in classification, particularly among former smokers and early-stage patients [129].

In a more recent study, Mitchell et al. [120] leveraged NGS to analyze miRNA profiles in EBC, confirming significant deregulation between lung cancer patients and healthy controls. Their findings highlighted the consistent upregulation of miR-155 and miR-22, alongside the downregulation of miR-9, miR-486, miR-34c, miR-206, and miR-503. Additionally, Let-7e was found to be upregulated, whereas miR-1 and miR-451 were reduced in cancerous samples. Notably, the study demonstrated that miRNA profiles in EBC closely resembled those in BAL, reinforcing the potential of EBC-derived miRNAs as non-invasive biomarkers for lung cancer diagnosis.

Notably, several of the cited studies—including those by Pérez–Sánchez et al. [126], Shi et al. [129], and Mitchell et al. [120]—were conducted in clinical or clinic-based case-control settings. These investigations analyzed EBC-derived miRNA signatures from lung cancer patients and healthy controls using genome-wide arrays or NGS platforms. The studies not only demonstrated diagnostic value but also explored correlations with histological subtype, disease stage, and patient prognosis. Such findings underline the translational relevance of EBC miRNA profiling and represent significant steps toward its integration in clinical diagnostics.

### 4.5. EBC Limitations for the Analysis of MiRNA Expression

The application of exhaled breath condensate for miRNA profiling faces substantial methodological limitations, particularly concerning data reproducibility. Even among studies examining lung cancers of similar histological types and stages, inconsistencies in the identified miRNA signatures are frequently observed. This variability is likely influenced by the complex composition of EBC, which is primarily composed of water vapor and contains aerosolized particles from different anatomical regions of the respiratory system, including the alveoli, bronchi, and oral cavity [130]. Therefore, EBC samples may incorporate nucleic acid material from both affected and unaffected pulmonary tissues, reducing biomarker specificity.

Another critical limitation is the typically low abundance of miRNAs in EBC, which often falls below the detection capabilities of standard molecular assays. Consequently, only miRNAs expressed at quantifiable levels, along with highly sensitive detection platforms, are suitable for robust biomarker discovery. Furthermore, the lack of a standardized dilution correction method complicates data normalization and comparison across studies. Additional confounding factors, such as variability in collection procedures, subject-related differences, and environmental influences, further compromise the reliability of miRNA measurements in EBC [131,132].

Moreover, many of the currently available studies are limited by small sample sizes, single-center designs, and a lack of validation in independent cohorts, which substantially hinders the generalizability and clinical applicability of their findings. Collectively, these challenges reflect the current developmental stage of EBC-based miRNA diagnostics and highlight the necessity for harmonized protocols, multicenter collaborations, and robust validation frameworks.

## 5. Conclusions

EBC analysis emerges as an innovative and promising approach for extracting miRNAs, offering a non-invasive and practical method potentially suitable for early lung cancer diagnosis. Recent studies have highlighted the relevance of miRNAs as sensitive and specific biomarkers, and the use of EBC in conjunction with advanced extraction methods such as NGS represents a significant technological advancement in this field. Unfortunately, many of them are limited by heterogeneity, small sample sizes, single-center designs, a variable miRNA yield from EBC, and a lack of validation in independent cohorts, which substantially hinders the generalizability and clinical applicability of their findings. These challenges reflect the current developmental stage of EBC-based miRNA diagnostics and highlight the necessity for harmonized protocols, multicenter collaborations, and robust validation frameworks.

However, further research is required to standardize collection and analytical protocols, improve result reliability, and integrate this technique into routine clinical workflows. Implementing this approach could revolutionize screening strategies and early diagnosis, opening new perspectives for improving the prognosis and management of lung cancer patients.

## Figures and Tables

**Table 1:** Advantages and limitations of EBC versus other liquid biopsy matrices.

Matrix	Advantages	Limitations
Exhaled Breath Condensate	- Completely non-invasive - High patient compliance - Reflects airway-specific processes - Suitable for repeated sampling	- Low miRNA concentration - High dilution (>99.9% water) - Lack of standardization - Contamination risk (oral/upper airway)
Plasma	- Minimally invasive - High biomarker concentration - Well-standardized collection and handling - Broad diagnostic use	- Low specificity for lung-related processes - Invasive for repeated use - Systemic signal dilution
Sputum	- Originates from the respiratory tract - Moderately high RNA yield - Non-invasive (when spontaneous)	- Variable sample quality - Possible oral contamination - Difficult to obtain from asymptomatic individuals
Bronchoalveolar lavage	- High local specificity - High RNA yield - Established protocols in clinical settings	- Invasive (requires bronchoscopy) - Low patient acceptability - Not suitable for routine screening

**Table 2:** Summary of Exhaled Breath Condensate (EBC) Collection Systems.

Reference	System	Advantages	Disadvantages
Konstantinidi et al. (2015) [61]	RTube collection system (Respiratory Research Inc., Austin, TX, U.S.)	- Rapid cooling mechanism—Commercially available—Enables unsupervised home-based sample collection	- Prolonged collection time (~15 min for 500 μL)—Potential analyte adsorption onto collection tube surfaces—Dilution effects in EBC
Piotrowski et al. (2010) [91]	ECoScreen I and ECoScreen II (Erich Jaeger GmbH, Hochberg, Germany) U.S.,	- Optional package for determining the total EBC	- Not portable—No control of condensation temperature (Eco1)
Izquierdo et al. (2006) [92]	Anacon (Biostec, Valencia, Spain)	- Usage on ventilated patients—Monitoring and control of temperature collection	- Lacks clinical validation
Cherchi et al. (2023) [93]	TurboDeccs (Medivac Srl, Parma, Italy)	- Disposable components—Monitoring and control of temperature collection—Portable—Optional package for determining the total EBC	- Multiple simultaneous collections not allowed
McCafferty et al. (2004) [64]	Temperature-selective collection approach	- Differentiates between dead space and deep lung air, reducing EBC dilution	- Complex collection setup—Lacks clinical validation
Balbi et al. (2007) [65]	Disposable sampling device for viral particles	- Efficient capture of viral particles in EBC—Minimal dilution with ambient air—Effective collection of ultrafine particles (<300 nm)	- Complex collection methodology—Absence of clinical validation
Effros et al. (2002) [66]	Facial mask with detachable medical adhesive	- Wearable design enhances user compliance—Facilitates pre-concentration of collected sample	- Extended collection time—Lacks clinical validation
Liu et al. (2007) [67]	Facial mask-based EBC collection	- Wearable and user-friendly—Short collection duration (~5 min for 500 μL)—Potential for sensor integration	- Lacks clinical validation—Ongoing clinical trials

**Table 4:** Lung cancer report by microRNA analysis from exhaled breath condensate samples.

Reference	Aims	Group	Method	miRNAs	Result	Interpretation
Mozzoni et al. (2013) [121]	To identify biomarkers that increase the likelihood of early detection of LC	NSCLC n = 54 Control n = 46 (26 nodules, seven bronchiectasis, 13 others: emphysema (n = 2), inflammatory outcomes (n = 2), aspiration pneumonia (n = 1), tuberculosis (n = 1), rhino-bronchial syndrome (n = 1), asthma with allergic rhinitis (n = 1), unspecified radiological alteration (n = 5)	qRT-PCR assay	miR-21 miR-486	Upregulated Downregulated	miR-21 was significantly upregulated and miR-486 was downregulated in LC as compared with the control group. miR-21 was reported as oncogenic, and miR-486 as a tumour-suppressor miRNA
Chen et al. (2016) [123]	To investigate the clinical significance of miRNA21 in patients with NSCLC	NSCLC n = 30 Control n = 30	qRT-PCR assay	miR-21	Upregulated	miR-21 was significantly upregulated in NSCLC as compared with the control group.
Ibrahim et al. (2017) [122]	To evaluate the role of miRNA-155 in the diagnosis, as well as the prognosis of LC	LC n = 15 Control n = 15	qRT-PCR assay	miR-155	Upregulated	miR-155 was upregulated in LC patients as compared with the control group. Reported oncogene and a potential biomarker for early LC detection, as well as for prognosis
Chen et al. (2020) [124]	To investigate the association between let-7 and NSCLC	NSCLC n = 30 Healthy control n = 30	qRT-PCR assay	Let-7	Downregulated	Levels of Let-7 were downregulated in NSCLC patients compared to healthy controls.
Xie et al. (2020) [125]	To investigate the expression level and clinical significance of serum and EBC miR-186 and IL-1β in NSCLC patients	NSCLC n = 62 Healthy control n = 60	qRT-PCR assay	miR-186	Downregulated	Levels of miR-186 decreased in the EBC of NSCLC patients compared with healthy controls.
Pérez-Sànchez et al. (2021) [126]	To assess the utility of EBC miRNAs as biomarkers of diagnosis, type of tumour, stage, invasion capacity, and prognosis	LC n = 21 Healthy donor n = 21	Genome-wide microarray	miR-6865-5p, miR-4707-5p, miR-451a, miR-1469, miR-4507, miR-6780a-5p, miR-668-5p, miR-6794-5p and miR-7855-5p miR-3921, miR-320a and miR-6777-5p	Upregulated Downregulated	Nine microRNAs (miR-6865-5p, miR-4707-5p, miR-451a, miR-1469, miR-4507, miR-6780a-5p, miR-668-5p, miR-6794-5p and miR-7855-5p) were upregulated in the EBC of LC patients. Three microRNAs (miR-3921, miR-320a and miR-6777-5p) were downregulated compared with healthy controls.
Faversani et al. (2021) [127]	Early detection of LC by profiling miRNA in EBC (non-invasive method)	Adenocarcinoma n = 14 Healthy controls n = 9	qRT-PCR array	miR-597-5p, miR-1260a	Upregulated	miR-597-5p and miR-1260a were upregulated in the EBC of lung adenocarcinoma patients compared with healthy controls.
Rai et al. (2023) [128]	To validate the clinical utility of miRNAs from EBC as non-invasive biomarkers for LC patients	LC n = 30 Healthy controls n = 30	qRT-PCR array	miR-31-3p, Let7i and miR-449c	Upregulated	Expression of miR-31-3p, Let7i, and miR-449c was found to be upregulated in EBC of LC patients compared with healthy controls
Shi et al. (2023) [129]	To develop and test an miRNA detection strategy by EBC for LC case–control discrimination	NSCLC n = 166 Case controls n = 185	qRT-PCR array	miR-21r, miR-33b, miR212	Upregulated	Adjusted logistic regression models identified exhaled miR-21, 33b, and 212 as overall case–control discriminants.
Mitchell et al. (2024) [120]	To target the LC population based on miRNAs carried by extracellular vesicles in EBC	NSCLC n = 6 Healthy controls n = 12	NGS analysis	let-7c, mir-155, miR-22, miR-378, miR-125b, miR-133a, miR-222, miR-210 miR9, miR-486, miR-34c, miR-206, miR-100, miR-503, miR-1, miR-451	Upregulated Downregulated	Tissue-specific enrichment of exhaled extra vesicles from EBC may help identify miRNAs whose deregulated expression correlates with LC.

NSCLC: non-small cell lung cancer; n: number; qRT-PCR: quantitative real time—protein chain reaction; miRNA: micro ribonucleic acid; LC: lung cancer; EBC: exhaled breath condensate; NGS: next-generation sequencing.

## Data Availability

All data generated as part of this study are included in the article.
